# Supplementary material for: Parallel processing of working memory and temporal information by distinct types of cortical projection neurons
Source: Nat Commun. 2021 Jul 16;12:4352. doi: 10.1038/s41467-021-24565-z (PMC8285375; doi:10.1038/s41467-021-24565-z)
Supplement: Supplementary file 1 — Supplementary Information [file 41467_2021_24565_MOESM1_ESM.pdf]

SUPPLEMENTARY INFORMATION

Parallel processing of working memory and temporal information by distinct types of cortical projection neurons

Jung Won Bae, Huijeong Jeong, Young Ju Yoon, Chanmee Bae, Hyeonsu Lee, Se-Bum Paik & Min Whan Jung

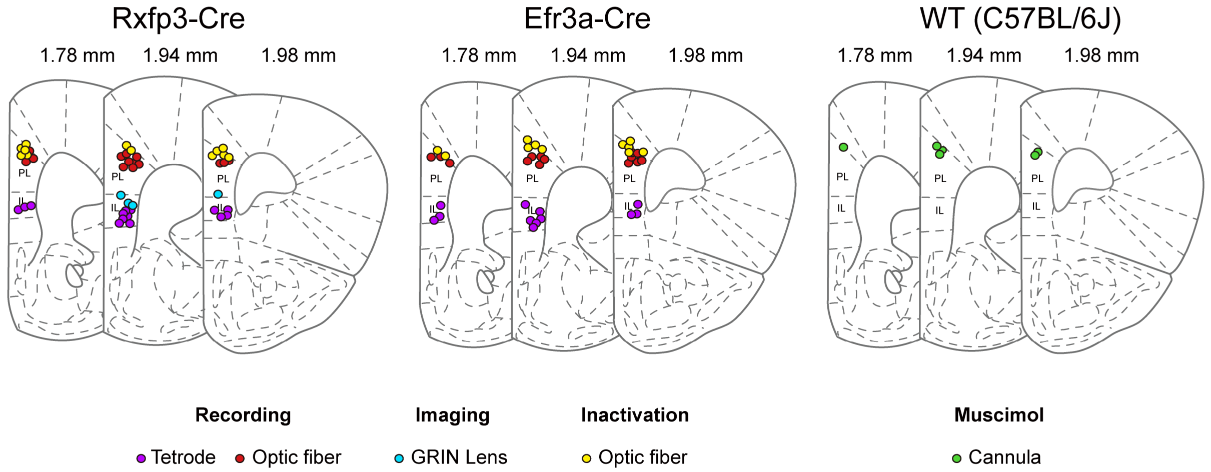

Supplementary Fig. 1 | Histological identification of cannula, optic fiber, tetrode, and GRIN lens locations. Shown are coronal section views of the mouse brain (left to right, 1.78, 1.94, and 1.98 mm anterior to bregma). Circles indicate locations of cannula tips used for muscimol infusion (green; WT), optical fiber tips used for optical tagging (red; Rxfp3-Cre and Efr3a-Cre), tetrode tips at the end of the final recording session for each mouse (purple, one representative tetrode for each mouse; Rxfp3-Cre and Efr3a-Cre), optical fiber tips used for inactivation of IT or PT neurons (yellow; Rxfp3-Cre and Efr3a-Cre), and tips of prism attached to GRIN lens used for calcium imaging of IT neurons (cyan; Rxfp3-Cre).

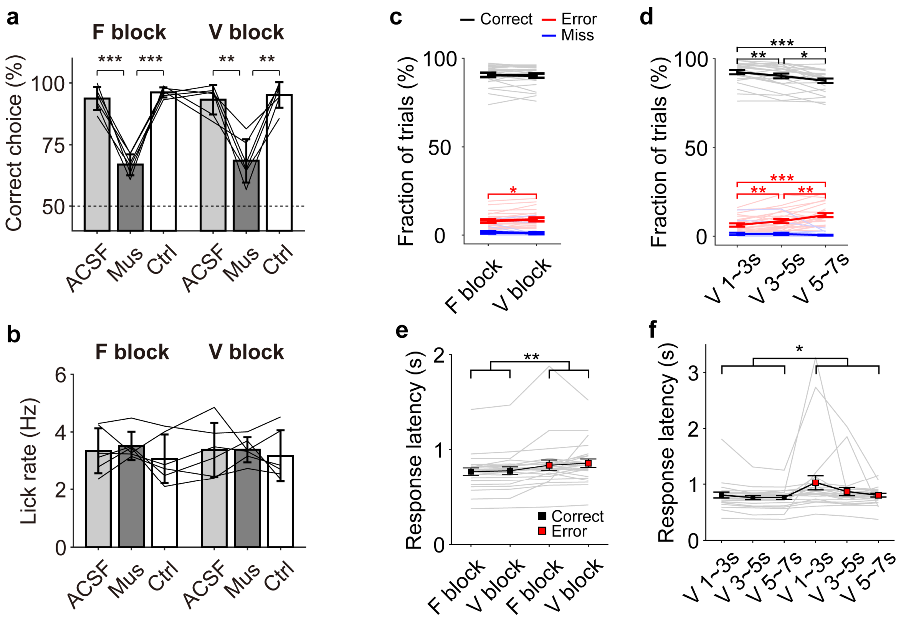

Supplementary Fig. 2 | Behavioral performance. a-b, Behavioral performance (% correct choices; **a**) and lick rate (**b**) of WT mice (n = 6) during the fixed-delay and variable-delay blocks (F block and V block, respectively) with (Mus) and without (ACSF and Ctrl) mPFC inactivation. The same format as in Fig. 1e, f. **c-f,** Behavioral performance and response latency of the mice used in the main physiological experiment (n = 24; 12 Efr3a-Cre and 12 Rxfp3-Cre mice that performed both fixed-delay and variable-delay tasks were pooled together; two Rxfp3-Cre mice that performed only the fixed-delay task were excluded from this analysis). **c,** Fractions of correct (black), error (wrong target choice; red), and miss (no choice; blue) trials during the fixed-delay and variable-delay blocks. **d,** Fractions of correct, error, and miss trials as a function of delay duration. Trials during the variable-delay block were grouped into three according to delay duration (1 ~ 3, 3 ~ 5, and 5 ~ 7 s). **e,** Response latencies (time until the first lick since delay offset) in correct (black) and error (red) trials of fixed-delay and variable-delay blocks. **f,** Response latencies in correct (black) and error (red) trials at different delay durations. *$p < 0.05$; **$p < 0.01$; ***$p < 0.001$ (a-b and e-f, two-way repeated measures ANOVA followed by Bonferroni's post-hot tests; c, paired *t*-test; d, one-way repeated measures ANOVA followed by Bonferroni's post-hoc tests). Thin lines, individual animal data; error bars, SEM across animals.

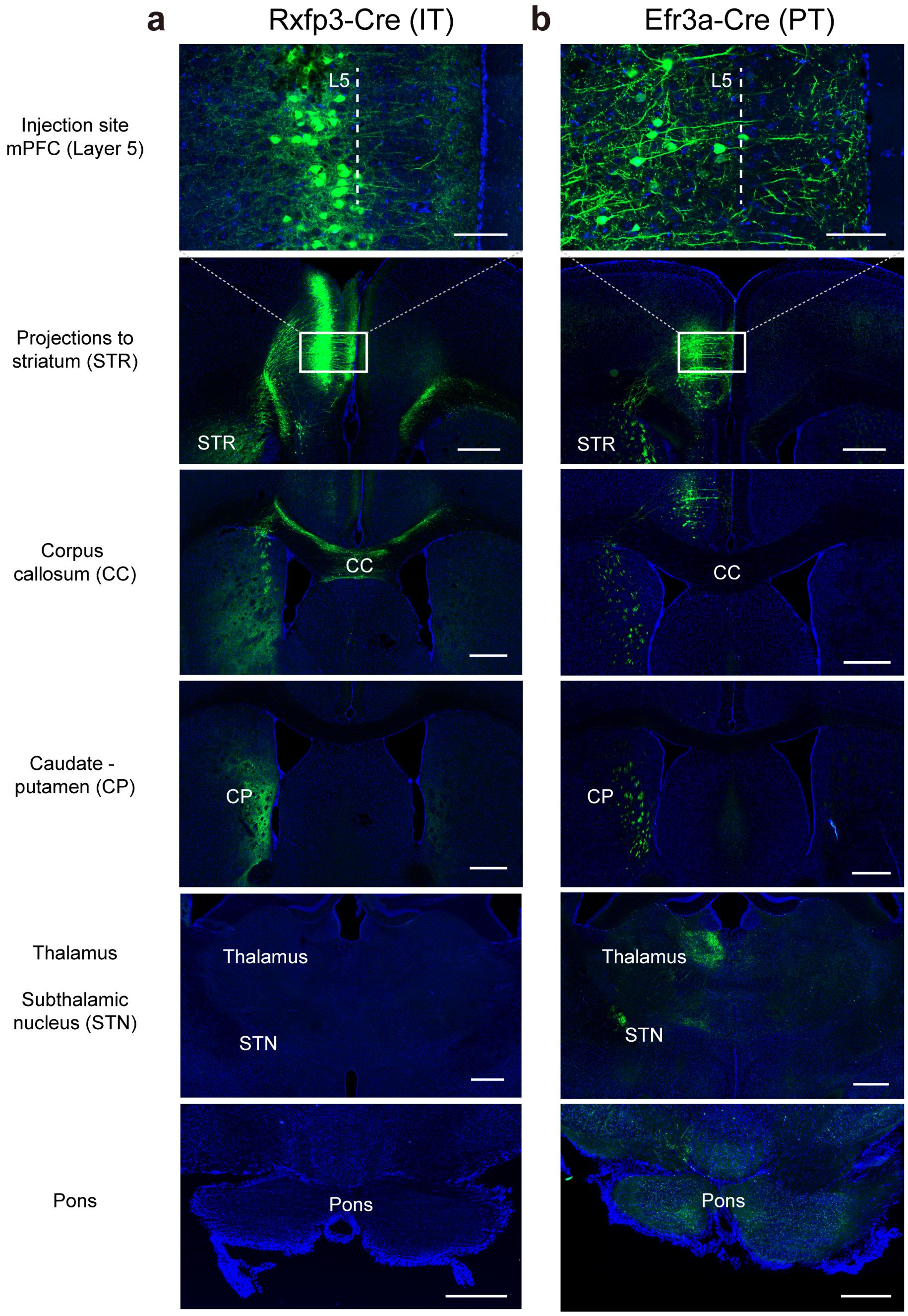

Supplementary Fig. 3 | Histological verification of IT and PT neurons. Shown are example coronal brain sections showing fluorescence signals in Rxfp3-Cre and Efr3a-Cre mice (representative of n = 2 and 4 experiments using Rxfp3-Cre and Efr3a-Cre mice, respectively). We injected AAV2/Ef1a-DIO-eYFP (Rxfp3-Cre mouse) or AAV2-CAG-FLEX-tdTomato (Efr3a-Cre mouse) unilaterally into the mPFC and performed histology four weeks later to examine the

distribution of the fluorescence signals. As expected, fluorescence expression was detected in the deep layers of the mPFC in both mice. It was additionally detected in the contralateral cortices, as well as in the ipsilateral and contralateral striatums of the Rxfp3-Cre mouse. In the Efr3a-Cre mouse, it was additionally detected in the ipsilateral striatum and other subcortical structures. **a,** In the Rxfp3-Cre mouse, the injection labeled mPFC layer 5 neurons (AAV2-eYFP, green, top two panels) that sent bilateral axonal projections to the striatum (STR, second to fourth panels) via the corpus callosum (CC, third panel), but not to the thalamus, subthalamic nucleus (STN) or pons (bottom two panels). This indicates selective labeling of deep-layer IT neurons. **b,** In the Efr3a-Cre mouse, the injection labeled mPFC layer 5 neurons that projected to the striatum, thalamus, and STN ipsilaterally (above pyramidal decussation), and to the pons bilaterally (below pyramidal decussation; AAV2-tdTomato, pseudo-colored green), indicating selective labeling of PT neurons. Dashed lines in the top panels indicate the border between layers 3 and 5. Scale bar, 100 μm (top panels) or 500 μm (the rest).

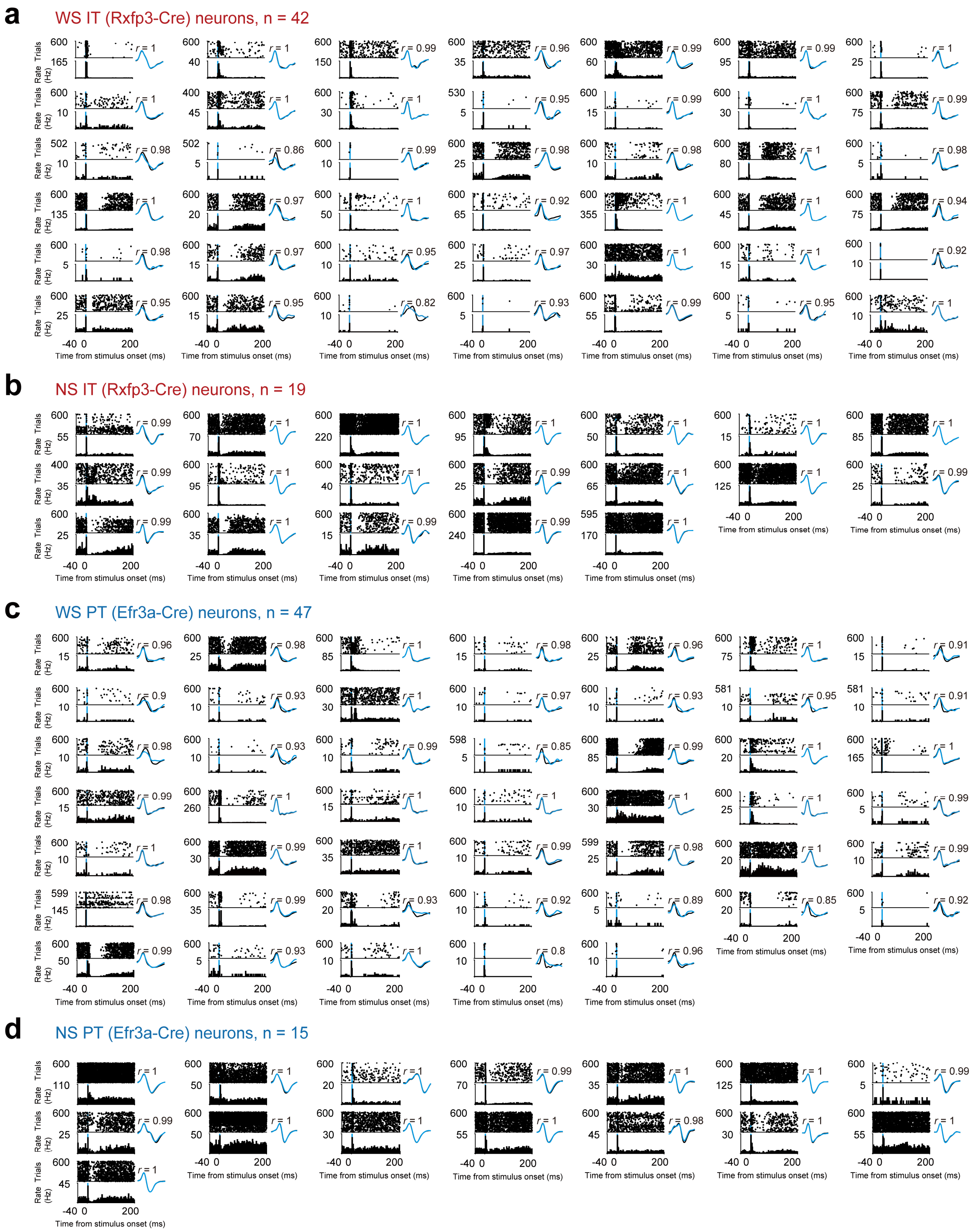

Supplementary Fig. 4 | Optogenetic identification of IT and PT neurons. Shown are the responses of optically tagged IT and PT neurons to laser stimulation (blue bars; 5 ms) recorded from Rxfp3-Cre (a, b) and Efr3a-Cre (c, d) mice, respectively. Optically tagged neurons classified

neither as WS nor NS neurons (n = 5 from Rxfp3-Cre mice) are excluded. Top, spike raster plots; each line is one trial and each tick mark represents a spike. Bottom, peri-stimulus time histograms. Insets, averaged waveforms of spontaneous (black) and optically driven (blue) spikes (duration, 1 ms; calibration for spike amplitude varies across neurons; *r*, correlation coefficient between the two waveforms).

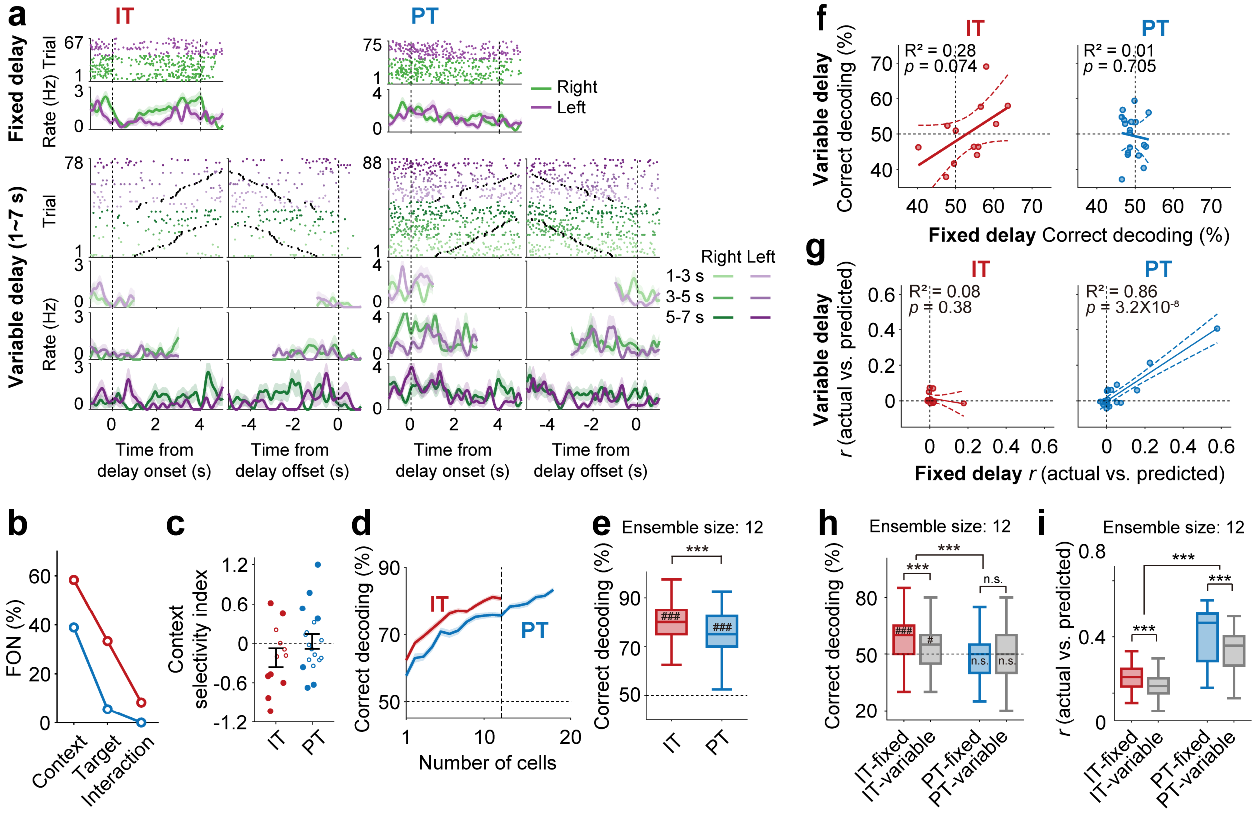

Supplementary Fig. 5 | Comparison of delay-period activity across fixed- and variable-delay conditions. a, Examples for IT and PT neuronal activity during the fixed- (top) and variable- (bottom) delay conditions. Neural responses during the variable-delay condition are shown twice aligning trials to delay onset (left; black circles denote delay offset) or offset (right; black circles denote delay onset), and spike density functions are shown separately for three different ranges of delay duration (more intense colors indicate longer delays). **b,** Fractions of neurons significantly responsive to behavioral context (fixed- versus variable-delay condition), target (left versus right lick port), and their interaction during the delay period. Mean firing rate during the delay period was subjected to two-way ANOVA. As shown, large fractions of neurons changed their delay-period activity across the fixed- and variable-delay conditions. **c,** Context selectivity index (positive and negative values denote higher delay-period activity in the fixed- and variable-delay blocks, respectively) was not significantly different from 0 (*t*-test, $p > 0.153$). **d-e,** Neural decoding of behavioral context. Behavioral context was decoded based on neuronal ensemble activity during the delay period using the SVM. Shown are decoding performance as a function of neuronal ensemble size (**d**) and mean decoding performance at ensemble size of 12 (**e**; verical dashed line in d). ###$p < 0.001$ (above chance level, *t*-test); ***$p < 0.001$ (IT versus PT, *t*-test). **f,** Neural decoding of target during the fixed- and variable-delay blocks. Decoding performance of IT neurons showed a trend for positive correlation across blocks. **g,** Neural decoding of time (correlation between actual and predicted time bins) during fixed- and variable-delay blocks. Decoding performance was significantly correlated across blocks for PT, but not IT, neurons. **h,**

Neural decoding of target under the fixed- and variable-delay conditions at ensemble size of 12 neurons. **i,** Neural decoding of time under the fixed- and variable-delay conditions at ensemble size of 12 neurons. #$p < 0.05$, ###$p < 0.001$ (above chance level, *t*-test); ***$p < 0.001$ (across groups, two-way mixed ANOVA followed by Bonferroni's post-hoc tests). Variable-delay neural data were analyzed up to 4 s using those neurons recorded in the sessions with ≥10 correct trials for each target (n = 12 IT and 18 PT neurons) and using those trials with delay durations ≥ 4 s. Circles in c, f, and g denote individual neuronal data; filled circles in c denote significant index (*t*-test, $p < 0.05$). Shading, SEM across trials (a), neurons (c) or 100 decoding iterations (d). Box plots (e-i) show median, interquartile range, and maximum and minimum within 1.5 interquartile range.

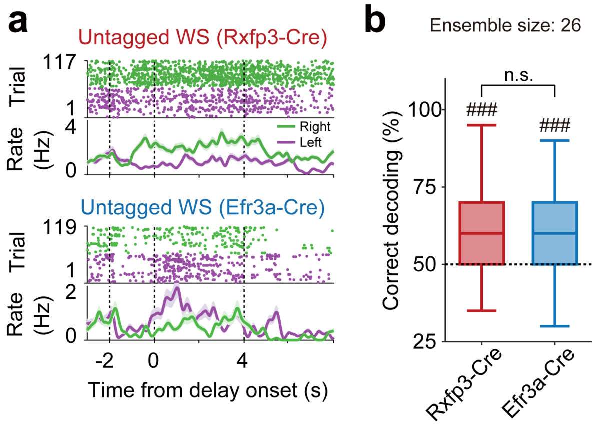

Supplementary Fig. 6 | Comparison of target signals carried by untagged WS neurons between Rxfp3-Cre and Efr3a-Cre mice. a, Example responses of untagged WS neurons during the fixed-delay block (correct trials only). **b,** Decoding target based on delay-period (4 s) ensemble activity of untagged WS neurons recorded from Rxfp3-Cre (red) or Efr3a-Cre (blue) mice (100 iterations using 26 randomly selected neurons). Boxplots (b) show median, interquartile range, and maximum and minimum within 1.5 interquartile range. ###$p < 0.001$ (above chance level, *t*-test); n.s., $p > 0.05$ (IT versus PT, *t*-test).

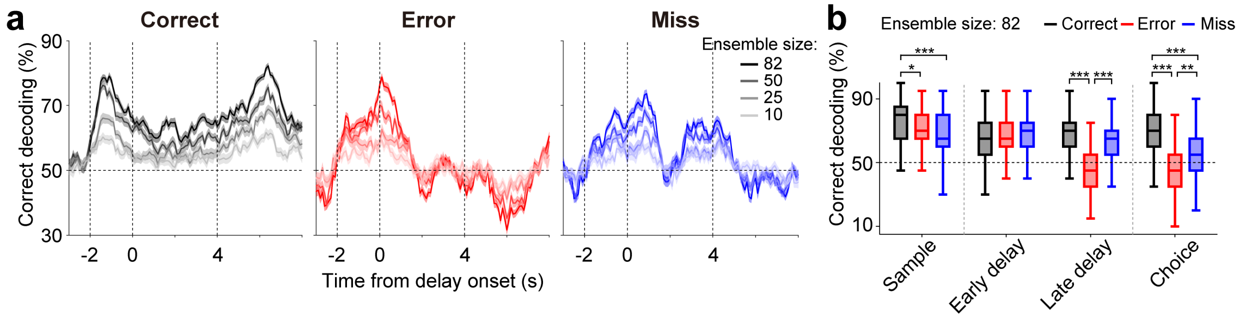

Supplementary Fig. 7 | Neural decoding of target differs between error and miss trials. Untagged WS neurons (n = 82) recorded in the sessions with ≥ 2 error and ≥ 2 miss trials for both targets were analyzed. Only correct trials were used as training data, and correct, error, and miss trials were used as test data. **a,** Temporal profiles of decoding performance (1-s sliding window advanced in 0.1 s steps) at different ensemble sizes. Shading, SEM across 100 decoding iterations. **b,** Decoding performances of correct, error, and miss trials at different phases of the task (ensemble size = 82). Sample phase, 2-s time period before delay onset; Early and late delay, first and second halves of the 4-s delay period; Choice phase, 2-s time period since delay offset. Box plots (b) show median, interquartile range, and maximum and minimum within 1.5 interquartile range. *$p < 0.05$, **$p < 0.01$, ***$p < 0.001$ (one-way repeated measures of ANOVA followed by Bonferroni's post-hoc tests).

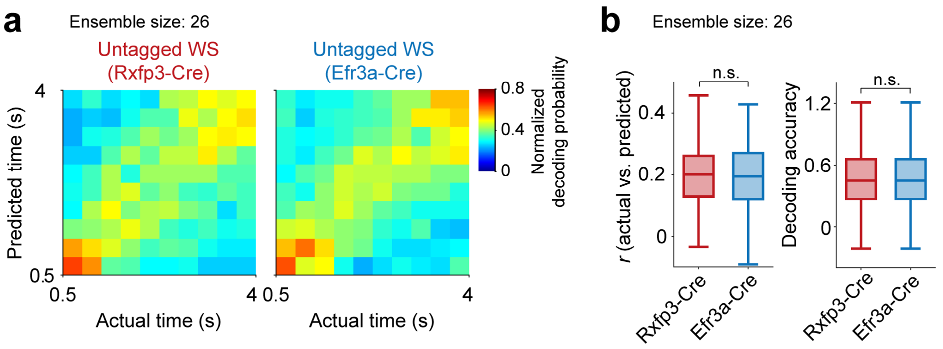

Supplementary Fig. 8 | Comparison of temporal information carried by untagged WS neurons between Rxfp3-Cre and Efr3a-Cre mice. a, Heat maps showing mean normalized decoding probabilities (actual versus predicted bins) using 26 randomly selected untagged WS neurons recorded from Rxfp3-Cre (left) or Efr3a-Cre (right) mice. **b,** Decoding performances (left, correlation between the actual and predicted bins; right, decoding accuracy; ensemble size, n = 26 neurons). n.s., $p > 0.05$ (t-test). The same format as in Fig. 6a, b. Box plots (b) show median, interquartile range, and maximum and minimum within 1.5 interquartile range.

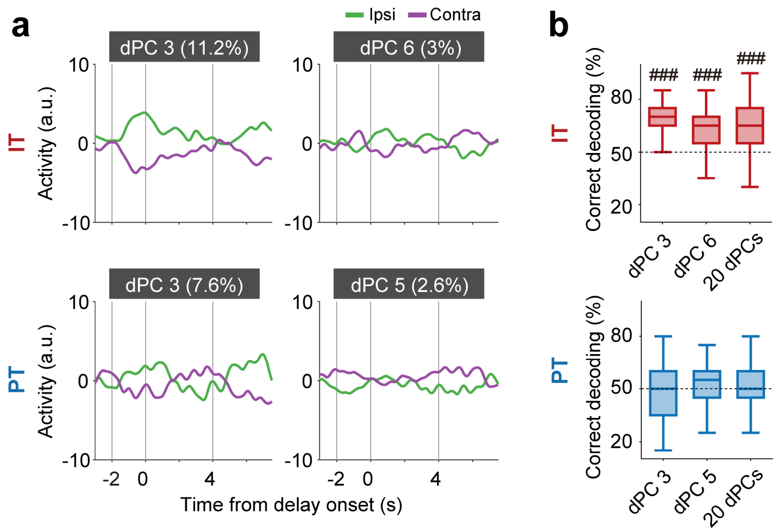

Supplementary Fig. 9 | Target-dependent dPCs of PT neurons do not carry significant target signals during delay period. a, Time courses of the top two target-dependent dPCs. Green and purple colors denote ipsilateral and contralateral target trials, respectively. **b,** Decoding performance using only the delay-period component (4 s) of each target-dependent dPC or 20 dPCs. The same sets of IT and PT neurons as in Fig. 6e-g (n = 26 and 31, respectively) were analyzed using 10 correct trials for each target. ###$p < 0.001$ (above chance level**,** *t*-test). These results indicate that target dependence of these PT neuronal dPCs is because of target-dependent neuronal activity before and/or after, but not during, the delay period. Box plots (b) show median, interquartile range, and maximum and minimum within 1.5 interquartile range.

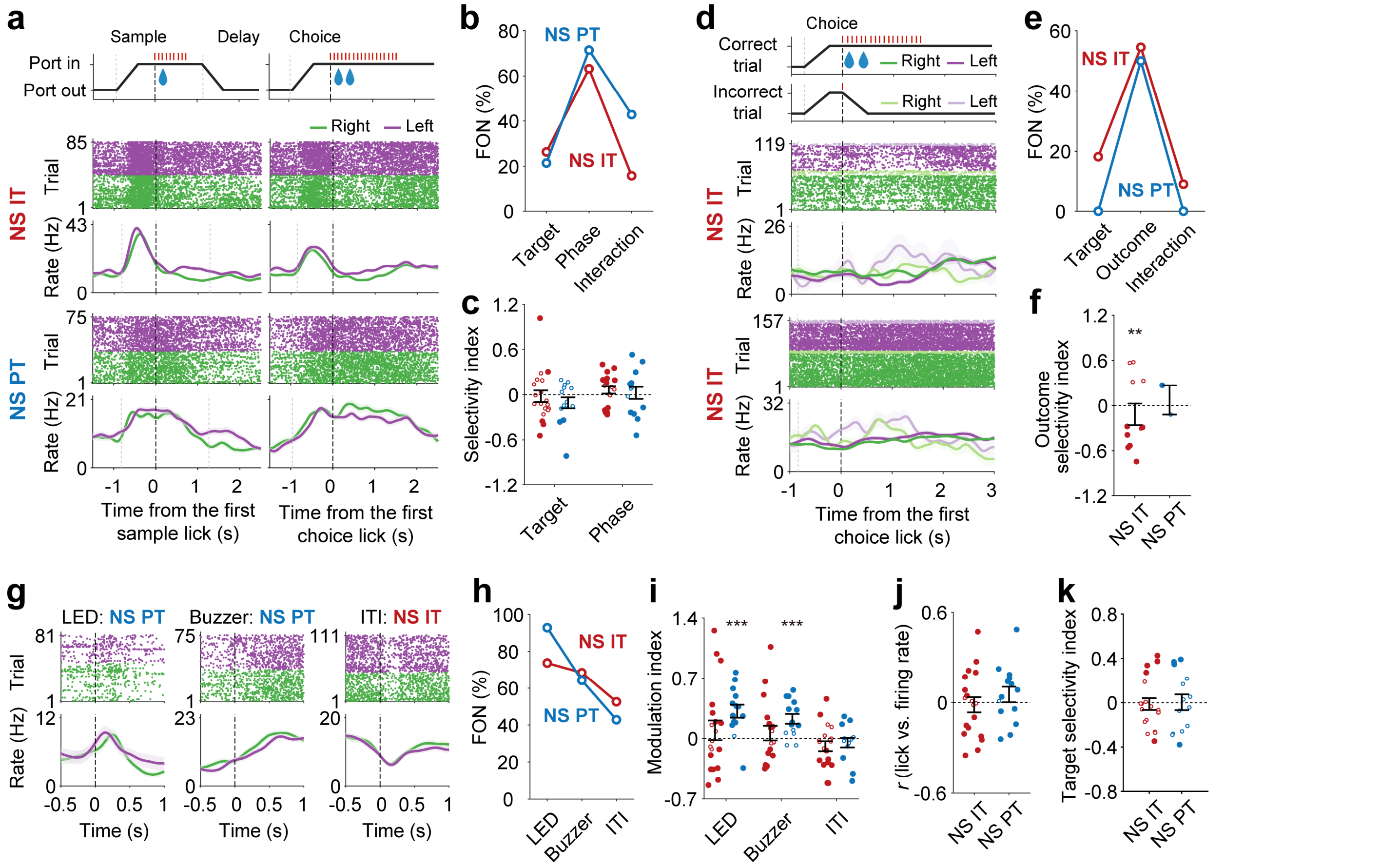

Supplementary Fig. 10 | Responses of NS IT and NS PT neurons to diverse task events. The same format as in Fig. 7. Only correct trials were analyzed (n = 19 NS IT and 14 NS PT neurons) except the analyses related to outcome-dependent neural activity (d-f; n = 11 NS IT and 2 NS PT neurons recorded in the sessions with ≥ 2 error trials for each target). Shading, SEM across trials (a, d and g). Error bars, SEM across neurons (c, f, and i-k).

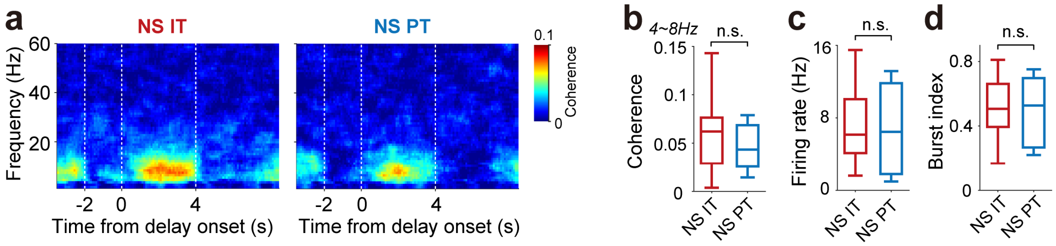

Supplementary Fig. 11 | Theta-frequency activity of optically-tagged NS neurons. a, Frequency-dependent spike-field coherence of NS IT (n = 18) and NS PT (n = 12) neurons in correct trials. **b-d,** Mean theta band spike-field coherences (b), firing rates (c) and burst indices (d) of NS IT and NS PT neurons during the delay period. The same formats as in Fig. 5d-g. Theta coherence (a, b), mean firing rate (c), and burst index (d) did not vary significantly between the cell types (NS IT versus NS PT, *t*-test). Box plots (b-d) show median, interquartile range, and maximum and minimum within 1.5 interquartile range.

Figures	Criteria for cell inclusion					Analyzed cells	
	All analyses	Error trial analysis	Miss trial analysis	LFP analysis	Variable-delay analysis		
	Firing rate ≥ 0.5Hz; ≥ 10 correct trials for each target	≥ 2 Error trials for each target	≥ 2 Miss trials for each target	< 1% of LFP signals reaching the ceiling	≥ 10 correct trial for each target under variable-delay condition	Cell type	Number of cells
Fig. 3, 6a-c, 6e-g, 7a-c, 7g-k, Fig. S9	O					WS IT	26
						WS PT	31
Fig. 9, Fig. S10a-c, S10g-k	O					NS IT	19
						NS PT	14
Fig. S6, S8	O					Untagged WS (Rxfp3-Cre)	392
						Untagged WS (Efr3a-Cre)	366
Fig. 4a-b, Fig. 6d	O	O				Untagged WS	365
Fig. 4d-g	O	O				IT	2,974 (95 ~ 451 per session)
Fig. 7d-f	O	O				WS IT	16
						WS PT	13
Fig. S10e-f	O	O				NS IT	11
						NS PT	2
Fig. 5b-h	O	O		O		Untagged WS	352
Fig. 5j-m	O			O		WS IT	25
						WS PT	31
Fig. S5	O				O	WS IT	12
						WS PT	18
Fig. S7	O	O	O			Untagged WS	82
Fig. S11	O			O		NS IT	18
						NS PT	12

Supplementary table 1 | Criteria for cell inclusion.
