# Supplementary material for: Parallel processing of working memory and temporal information by distinct types of cortical projection neurons
Source: Nat Commun. 2021 Jul 16;12:4352. doi: 10.1038/s41467-021-24565-z (PMC8285375; doi:10.1038/s41467-021-24565-z)
Supplement: Supplementary file 3 — Description of Additional Supplementary Files [file 41467_2021_24565_MOESM3_ESM.pdf]

Description of Additional Supplementary Files

File Name: Supplementary Movie 1
Description: Example trials during a 4-s fixed delay session in the order of leftcorrect, left-error, and miss trials.
