# Supplementary material for: Parallel processing of working memory and temporal information by distinct types of cortical projection neurons
Source: Nat Commun. 2021 Jul 16;12:4352. doi: 10.1038/s41467-021-24565-z (PMC8285375; doi:10.1038/s41467-021-24565-z)
Supplement: Supplementary file 5 — Reporting Summary [file 41467_2021_24565_MOESM5_ESM.pdf]

nature research

Corresponding author(s): Min Whan Jung

Last updated by author(s): Jun 14, 2021

Reporting Summary

Nature Research wishes to improve the reproducibility of the work that we publish. This form provides structure for consistency and transparency in reporting. For further information on Nature Research policies, see our Editorial Policies and the Editorial Policy Checklist.

Statistics

For all statistical analyses, confirm that the following items are present in the figure legend, table legend, main text, or Methods section.

n/a	Confirmed	
☐	☒	The exact sample size (n) for each experimental group/condition, given as a discrete number and unit of measurement
☐	☒	A statement on whether measurements were taken from distinct samples or whether the same sample was measured repeatedly
☐	☒	The statistical test(s) used AND whether they are one- or two-sided<br>*Only common tests should be described solely by name; describe more complex techniques in the Methods section.*
☒	☐	A description of all covariates tested
☐	☒	A description of any assumptions or corrections, such as tests of normality and adjustment for multiple comparisons
☐	☒	A full description of the statistical parameters including central tendency (e.g. means) or other basic estimates (e.g. regression coefficient) AND variation (e.g. standard deviation) or associated estimates of uncertainty (e.g. confidence intervals)
☐	☒	For null hypothesis testing, the test statistic (e.g. F, t, r) with confidence intervals, effect sizes, degrees of freedom and P value noted<br>*Give P values as exact values whenever suitable.*
☒	☐	For Bayesian analysis, information on the choice of priors and Markov chain Monte Carlo settings
☒	☐	For hierarchical and complex designs, identification of the appropriate level for tests and full reporting of outcomes
☐	☒	Estimates of effect sizes (e.g. Cohen's d, Pearson's r), indicating how they were calculated

Our web collection on statistics for biologists contains articles on many of the points above.

Software and code

Policy information about availability of computer code

Data collection	Neural data, including single unit and LFP signals, were collected using the Cheetah data acquisition system (Neuralynx, MT, USA). Putative single units were isolated using the MClust Software (version 3.5B.03, http://redishlab.neuroscience.umn.edu/MClust/MClust.html). The isolated units were classified using the 'fitmdist' function (expectation-maximization algorithm) of MATLAB. Theta frequency signals were extracted by filtering LFP signals between 4 and 8 Hz using EEGLAB toolbox (finite impulse response filtering; eegfilt function; https://sccn.ucsd.edu/eeglab/index.php) (Delorme and Makeig, 2004). The spike-LFP coherogram was calculated using the function "cohgramc" in the Chronux toolbox (version 2.12 v02). Calcium data were collected using nVista microscope. Spatial downsampling (x 1/4) and motion correction of calcium imaging data were performed using Inscopix Data Processing Software (version 1.3.1). The processed video was exported in TIFF format and analyzed with the CNMF-E algorithm to extract single unit signals.
Data analysis	Data were analyzed using MatLab (2017a). Adobe Illustrator CC 2017 was used in the production of data figures. All relevant codes supporting the present study are available upon reasonable request to the Lead Contact, Min Whan Jung .

For manuscripts utilizing custom algorithms or software that are central to the research but not yet described in published literature, software must be made available to editors and reviewers. We strongly encourage code deposition in a community repository (e.g. GitHub). See the Nature Research guidelines for submitting code & software for further information.

Data

Policy information about availability of data

All manuscripts must include a data availability statement. This statement should provide the following information, where applicable:

- Accession codes, unique identifiers, or web links for publicly available datasets
- A list of figures that have associated raw data
- A description of any restrictions on data availability

The neural and behavioral datasets used in this study are available at: https://www.dropbox.com/sh/y520iwmg8kly1w7/AAAq6umIPZIlJgJmuzK_kZ1Fa?dl=0. Source data for main figures 1~10 and supplementary figures S2 and S5~S11 are provided with this paper. Additional relevant data are available upon reasonable request to the Lead Contact, Min Whan Jung (mwjung@kaist.ac.kr).

Field-specific reporting

Please select the one below that is the best fit for your research. If you are not sure, read the appropriate sections before making your selection.

☒ Life sciences ☐ Behavioural & social sciences ☐ Ecological, evolutionary & environmental sciences

For a reference copy of the document with all sections, see nature.com/documents/nr-reporting-summary-flat.pdf

Life sciences study design

All studies must disclose on these points even when the disclosure is negative.

Sample size	Sample sizes were determined based on the published studies that addressed discharge characteristics of optically-tagged neurons in the mouse prefrontal cortex (Kim H. et al., 2016, Kim D. et al., 2016, Jeong H. et al., 2020).
Data exclusions	No animals were excluded from the analysis. Single neurons recorded in the sessions with insufficient trials (< 10 correct trials for both targets) or with low firing rates(< 0.5 Hz) in a given analysis window were excluded from all analyses. Additionally, in the analysis of error and miss trials, those neurons recorded in the sessions with less than 2 error and 2 miss trials for both targets were excluded. In the LFP analysis, the sessions with ≥ 1% of LFP signals reaching the ceiling were excluded. The criteria for cell exclusion were pre-established and are summarized in Supplementary Table 1.
Replication	All experiments were replicated at least three times.
Randomization	Animals were not randomized due to the necessity of a genetic construct (Rxfp3-Cre, Efr3a-Cre mice). Neurons were selected randomly for a given size of neuronal ensemble decoding. Trials were selected randomly for optogenetic modulation. Omission trials were selected randomly in the peak procedure task.
Blinding	The experimenters were not blind to subject groups because the knowledge of experimental conditions was required during data collection and evaluation.

Reporting for specific materials, systems and methods

We require information from authors about some types of materials, experimental systems and methods used in many studies. Here, indicate whether each material, system or method listed is relevant to your study. If you are not sure if a list item applies to your research, read the appropriate section before selecting a response.

Materials & experimental systems

n/a	Involved in the study	
☐	☒	Antibodies
☒	☐	Eukaryotic cell lines
☒	☐	Palaeontology and archaeology
☐	☒	Animals and other organisms
☒	☐	Human research participants
☒	☐	Clinical data
☒	☐	Dual use research of concern

Methods

n/a	Involved in the study	
☒	☐	ChIP-seq
☒	☐	Flow cytometry
☒	☐	MRI-based neuroimaging

Antibodies

Antibodies used	Primary antibody<br>Rabbit polyclonal anti-PV , Abcam, Cat#Ab11427, RRID: AB_298032 "https://www.abcam.com/parvalbumin-antibody-ab11427.html"<br><br>Secondary antibody

	Alexa568-conjugated anti-rabbit IgG, Molecular probe, Cat#A11011, RRID: AB_143157 "https://www.abcam.com/ASAP1--DDEF1-antibody-ab11011.html"
Validation	These are all well characterized commercial antibodies. The specificities of the primary and secondary antibodies were validated by the manufacturers. Validation profiles for all antibodies can be found in the following sites (Primary, https://www.abcam.com/parvalbumin-antibody-ab11427.html; Secondary, https://www.abcam.com/ASAP1--DDEF1-antibody-ab11011.html).

Animals and other organisms

Policy information about studies involving animals; ARRIVE guidelines recommended for reporting animal research

Laboratory animals	C57BL/6J, Ai9 tdTomato, Rxfp3-Cre and Efr3a-Cre male and female mice were used. All mice were aged 8-12 weeks at the time of surgery. Mice were single-housed after surgery under a 12 hr light/dark cycle (temperature, 19 ~ 23C; humidity, 45 ~ 55%).
Wild animals	No wild animals were used in this study.
Field-collected samples	No field samples were collected for analysis in this study.
Ethics oversight	All animal care and experimental procedures were performed in accordance with protocols approved by the directives of the Animal Care and Use Committee of Korea Advanced Institute of Science and Technology (approval number KA2020-10).

Note that full information on the approval of the study protocol must also be provided in the manuscript.
